# Splenic siderotic nodules in patients with liver cirrhosis

**DOI:** 10.3892/etm.2013.1135

**Published:** 2013-05-31

**Authors:** HUANG-QING OUYANG, ZUO-JIONG GONG, YUN-FEI ZHA, CHANG-SHENG LIU, ZHAO-HUI YANG

**Affiliations:** 1 Departments of Radiology, Renmin Hospital of Wuhan University, Wuhan, Hubei 430060, P.R. China; 2 Infectious Diseases, Renmin Hospital of Wuhan University, Wuhan, Hubei 430060, P.R. China

**Keywords:** spleen, siderotic nodules, magnetic resonance imaging, liver cirrhosis, hypersplenism, thrombocytopenia

## Abstract

The aim of this study was to investigate the interrelation between splenic siderotic nodules, hypersplenism and liver function in patients with liver cirrhosis. The splenic enhanced susceptibility-weighted angiography (ESWAN) and conventional magnetic resonance images of 33 patients with liver cirrhosis were retrospectively studied and the ESWAN images were graded. The distribution and prevalence of the image grades for patients with and without hypersplenism were evaluated. In addition, the splenic volume and the distribution of Child-Pugh and albumin scores were compared between patients with and without siderotic nodules, and the correlation between splenic volume and the ESWAN image grades were evaluated in the patients with siderotic nodules. The ESWAN images revealed splenic siderotic nodules in 24 patients. The distribution and prevalence of the ESWAN image grades were demonstrated to be significantly different (P<0.001) between patients with and without hypersplenism. Furthermore, significant differences were observed between patients with and without siderotic nodules with regard to splenic volume and the distribution of Child-Pugh and serum albumin scores (P<0.001). No significant correlation was demonstrated between splenic volume and the ESWAN image grades (P>0.05). In conclusion, a higher prevalence of splenic siderotic nodules (72.7%) was observed using the ESWAN sequence, in comparison with results from previous studies, obtained using the T1-spoiled gradient echo sequence. The presence of splenic siderotic nodules was consistent with the occurrence of hypersplenism and was interrelated with reserved liver function.

## Introduction

Splenic siderotic nodules, also known as Gamna-Gandy bodies (GGBs), are small granuloma-like nodules that most frequently occur within the spleen. GGBs were first observed in a patient with biliary cirrhosis and were described by the French physician, Charles Gandy (1872–1943), in 1905. The nature of the GGBs was further investigated in 1921 by the Italian pathologist, Carlo Gamna (1866–1950). GGBs have been revealed to measure a few millimeters in size and be composed of hemosiderin, calcium and fibrous tissue, with a crystal structure of CaPO_4_.FeOH. The iron inclusions favor the deposition of calcium salts (1,2).

There have been relatively few studies concerning the imaging of siderotic nodules in the spleen, although due to the hemosiderin inclusion within the nodules, it has been suggested that gradient-echo (GRE) magnetic resonance imaging (MRI) sequence may be more sensitive to this type of nodule than alternative imaging modalities (3–7). In the past decade, a novel MRI sequence, enhanced-susceptibility weighted angiography (ESWAN), has been widely used in clinical practice (8,9). Due to its sensitivity to the susceptibility differences between substances, such as deoxyhemoglobin, iron and calcium, this new sequence is able to enhance the susceptibility contrast between tissues and highlight lesions, such as hemorrhages and hemosiderin. The sequence has been predominantly utilized for brain imaging, although other applications have also been described (10–15). In 2011, a new 3.0 T MRI scanner was installed in the Department of Radiology, Renmin Hospital of Wuhan University (Wuhan, China) and the ESWAN sequence was utilized for the detection and assessment of liver siderotic nodules and iron deposition in patients with liver cirrhosis, in addition to being used for brain imaging. Following the observation of abnormal signals (siderotic nodules) in the spleen, a number of patients were retrospectively reviewed, the MRI appearances, liver function and blood cell counts were studied and the correlation between splenic siderotic nodules and hypersplenism was discussed. Furthermore, the interrelation between liver function, thrombocytopenia and splenomegaly and the formation of splenic siderotic nodules in patients with liver cirrhosis was evaluated. To the best of our knowledge, there have been no previous investigations into the interrelations between splenic siderotic nodules and the clinical data from patients with liver cirrhosis or into the application of the ESWAN sequence for splenic imaging.

## Materials and methods

### Patients

Thirty-three patients with liver cirrhosis, who were inpatients of the Department of Infectious Diseases (Renmin Hospital of Wuhan University), were retrospectively studied from May, 2011 to February, 2012. Patients with additional hematological diseases and hemochromatosis were excluded from the study. Fifteen healthy individuals, including four females and 11 males, with ages ranging from 35 to 68 years (mean age, 55 years) were then recruited as controls between January and February, 2012. The study was reviewed and approved by the Medical Ethics Committee of the Renmin Hospital of Wuhan University and informed consent was obtained from all participants. Patient data, including demographic details, hemoglobin levels, and white cell, red cell and platelet counts were evaluated. Liver function was assessed using the Child-Pugh scoring system. Hypersplenism was defined as splenomegaly with a platelet count of <150,000 /mm^3^ and/or a white cell count of <3,500 /mm^3^. Splenomegaly was defined as the craniocaudal length of the spleen measuring >12.0 cm (the accepted value for adults in the department). The splenic volume of patients was measured using the MRI post-processing workstation tool.

### MRI technique

MRI was performed using a Signa HDxt 3T scanner (GE Healthcare, Wasukesha, WI, USA) with an HD cardiac coil (GE Healthcare). The ESWAN sequence for the abdomen was an axial two-dimensional (2D) multi-echo gradient-echo sequence with six-echoes. The imaging parameters were as follows: Field of view, 35 cm; matrix, 320×288 pixels; repetition time (TR), 50 msec; echo times (TEs), 2.9, 8.0, 13.0, 18.0, 23.1 and 28.1 msec, respectively; flip angle, 20°; thickness, 5.0 mm and thickness gap, 20 mm. Six image slices of the spleen were obtained, with an acquisition time of 45 sec. The acquisition time was split into three consecutive blocks, with each block including a breath-holding period of 15 sec, followed by the acquisition of the data at the end-expiration. During each block, two image slices of the spleen were obtained, with each slice including 12 individual images (six raw magnitude and six corresponding phase images, respectively). The corrected phase and merged magnitude images were obtained using the post-processing station (GE Advantage Workstation 4.4, GE Healthcare).

Conventional sequences included the axial fat-suppressed fast spin-echo T2-weighted image (FS-FSE T2WI), with a TE of 90 msec and a TR of 6,316 msec; the coronal single-shot fast spin-echo T2-weighted image (SSFSE-T2WI), with a TE of 66.8 msec and a TR of 1,946 msec and the axial spoiled gradient-echo T1-weighted image (SPGR-T1WI), with a TE of 2.4 msec and a TR of 235 msec. The matrix was 320×224 pixels, while the thickness was 6.0 mm and the thickness gap was 2.0 mm.

### Imaging analysis

The images of the spleen obtained using the ESWAN sequence and conventional MRI were reviewed in consensus by two radiologists who were experienced in abdominal MRI imaging and who were blinded to the patient history. The cases that were considered to have ambiguous abnormal signals by the two reviewers were categorized into the normal group. If the reviewers expressed differing opinions, a third reviewer was consulted.

On the images obtained using ESWAN, signals that were lower than those for normal spleen parenchyma and similar to the spine in the same imaging slice were defined as dark signals. The reviewers were asked to grade the abnormal signals in the spleen into one of three categories (16): Grade A, normal, i.e. no dark signal present; grade B, scattered dark signals, with <10 in one slice; grade C, numerous dark signals, with >10 in one slice.

### Statistical analysis

Data analyses were performed using SPSS software, version 17.0 (SPSS, Inc., Chicago, IL, USA). P<0.05 was considered to indicate a statistically significant difference. The patients were classified into hypersplenism and non-hypersplenism subgroups. The distribution of the splenic ESWAN image grades across the two subgroups was evaluated using a Mann-Whitney U Test, while the prevalence of the grade C images was evaluated using a Fisher’s exact test. The application of the grade C images for the diagnosis of hypersplenism was assessed, and the sensitivity and specificity were calculated, respectively.

A comparison between the splenic volumes in patients with and without siderotic nodules, according to the ESWAN images, was performed using a t-test, while the distribution of the Child-Pugh and serum albumin scores (calculated according to the Child-Pugh scoring system) were evaluated using a Mann-Whitney U Test. With regard to the patients with siderotic nodules, the correlation between splenic volume and the grades of the ESWAN images was evaluated using Spearman’s rho.

## Results

### MRI appearance

**Figure 1.**
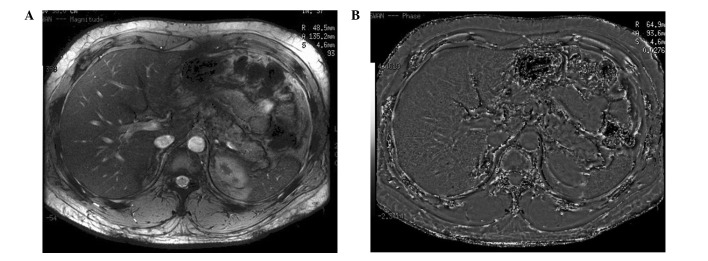
(A) Abdominal susceptibility-weighted magnitude image of a healthy control subject. The spleen has a normal appearance. (B) Abdominal susceptibility-weighted corrected phase image of a healthy control subject. No dark dots or nodules are present in the spleen.

**Figure 2.**
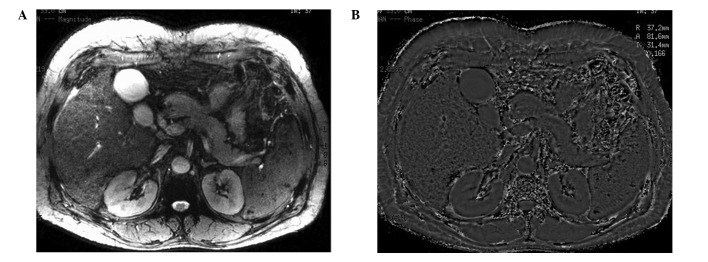
(A) Abdominal enhanced susceptibility-weighted angiography (ESWAN) magnitude image of a patient with liver cirrhosis. Numerous dark nodules were detected in the spleen. (B) Abdominal ESWAN-corrected phase image of the same patient with liver cirrhosis. Numerous dark nodules were detected in the spleen, corresponding with those on the magnitude image.

**Figure 3.**
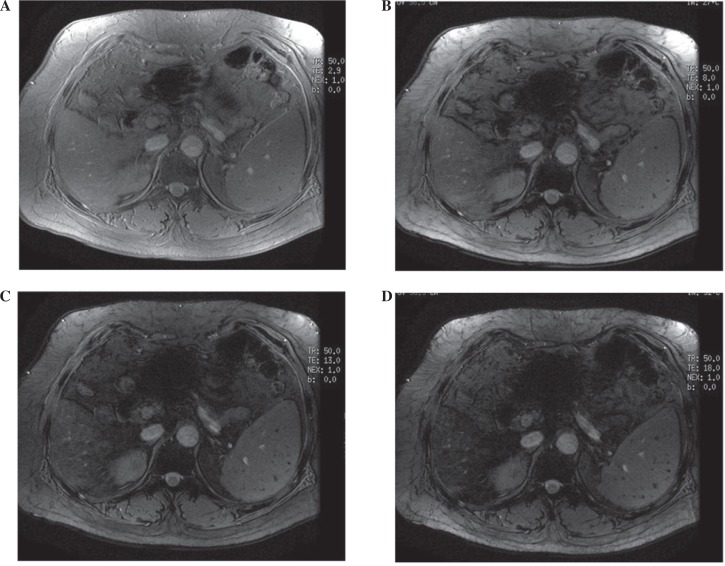
Sequential raw magnitude images of a patient with echo times (TEs) of (A) 2.9 msec, (B) 8.0 msec, (C) 13.0 msec and (D) 18.0 msec, respectively. The dark nodules are distorted on the short TE image and conspicuous on the long TE image.

No splenic signal abnormalities were observed on the ESWAN images from the healthy control subjects (Fig. 1). With regard to the 33 patients, speckled dark signals in the spleen were observed on the phase and magnitude ESWAN images in 24 cases (72.7%) (Fig. 2), while normal splenic signals were observed for the remaining nine cases. On the raw magnitude images, the dark lesions in the spleen were more conspicuous on the long TE images than on the short TE images (Fig. 3). With the conventional MRI, three cases (9.1%) showed splenic dark signals with the SPGR-T1WI sequence, while only one case (3.0%) demonstrated splenic dark signals with the TSE-T2WI sequence. The magnitude of the splenic dark signals was approximately a few millimeters and the distribution of the signals was random.

The patient data, the incidences of splenomegaly and hypersplenism and the grades of the splenic ESWAN images are shown in Table I. Three patients were observed to have a normal spleen size and did not exhibit any splenic dark signals. Of the 16 patients with hypersplenism, there were 14 grade C splenic ESWAN images, two grade B images and no grade A images. Of the 14 patients without hypersplenism, but with splenomegaly, there were eight grade B images and six grade A images. However, there were no grade C images.

### Statistical results

There were significant differences in the distribution of the grades of the splenic ESWAN images (P<0.001) between the hypersplenism and non-hypersplenism subgroups (Table II). Furthermore, there was a significantly different prevalence of the grade C images (P<0.001) (Table III) in the hypersplenism, compared with the non-hypersplenism, subgroup. The diagnostic sensitivity of the grade C images was 87.5%, while the specificity was 100%.

The splenic volume of the patients with siderotic nodules was significantly larger than those without the nodules (579.06±300.85 versus 323.74±179.10 ml, respectively; P<0.05). However, splenic volume did not demonstrate any significant correlation with the grades of the ESWAN images in the patients with siderotic nodules (r=0.319, P>0.05).

There were significant differences in the distributions of the Child-Pugh (Table IV) and serum albumin (Table V) scores (P<0.001 for each) between patients with and without siderotic nodules.

## Discussion

The ESWAN sequence includes magnitude and phase imaging. The magnitude imaging that was used in the present study was a type of T2*WI sequence. This is most sensitive to local field inhomogeneities, resulting in the phase changes with substances of different susceptibility. The phase imaging provides the phase shift information of the substances and is able to differentiate between iron and calcium due to their opposing susceptibilities. The former appears as a dark signal, while the latter results in a high signal on phase images. In this study, the lesions of the spleen were revealed to be small (<1 cm in size), with the appearance of dark signals (signal-voids) on all the sequences observed, although they were particularly conspicuous with the ESWAN sequence. It was observed that the dark signals in the spleen became darker and more conspicuous as the TE was increased on the raw magnitude images, which was consistent with the nature of the siderotic nodules (5–7,17). The nodules did not appear as high signals on the phase images in the study, which may be attributed to iron deposition being favored over the deposition of calcium or to a lack of calcium deposition within the nodule.

One factor that was not fully elucidated was whether the splenic siderotic nodules on the MRI images in the present study were identical to the GGBs described in previous studies. Splenic GGBs have been described to appear in a number of conditions, including chronic hemolysis, portal hypertension, sickle cell anemia (SCA), leukemia and lymphoma. It has been suggested that GGBs derive from red cell breakdown, and are a chronic, late event occurring following hemorrhage and red cell destruction (2). A histological study on splenic GGBs in patients with SCA demonstrated that GGBs were only detected by microscopy in patients >4 years of age, and that patients younger than this did not develop GGBs. Furthermore, hemosiderin deposits were observed in all patients, whereas microscopy revealed the presence of GGBs in 58% of cases (2). Hemosiderin deposition, old hemorrhages and GGBs may coexist and correspond with the different stages of hemolysis diseases, as well as liver cirrhosis, with hemorrhage and hemosiderin occurring in the early stages and GGBs appearing later. However, since all three are detected by a signal-void on MRI, due to their iron content, the differentiation may depend on histology. Therefore, the present study referred to siderotic nodules rather than GGBs.

Based on the images obtained using the ESWAN sequence, the prevalence of splenic siderotic nodules in patients with liver cirrhosis in the present study was 72.7%, which was higher than the prevalence observed in previous studies (9–13%), obtained using the T1-SPGR sequence. However, the results obtained with the T1-SPGR sequence in the present study were consistent with those of the previous studies (3,4,7,18). It was suggested that the ESWAN sequence was superior to the T1-SPGR sequence in the detection of siderotic nodules in the spleen.

Hypersplenism frequently occurs in patients with liver cirrhosis and portal hypertension. It is an independent risk factor for variceal bleeding, spontaneous bacterial peritonitis and death in patients with cirrhosis (19). Hypersplenism is diagnosed on the basis of clinical data that include splenomegaly with thrombocytopenia (cytopenia). However, the detection of splenomagely alone by imaging modalities is not representative of hypersplenism. The present study revealed that the presence of splenic siderotic nodules corresponded with the occurrence of hypersplenism. Since grade C images were only observed in patients with hypersplenism, the occurrence of these images may be applied to the diagnosis of hypersplenism. This diagnostic method was demonstrated to have desirable sensitivity and specificity.

It has been suggested that splenic siderotic nodules in patients with cirrhosis may be attributed to portal hypertension. It is possible to detect certain secondary signs of portal hypertension, such as portal widening, splenomegaly and variceal formation. In the present study, splenic siderotic nodules were only observed in patients with splenomegaly, which was consistent with previous invetigations (7,18). The splenic volume of the patients with siderotic nodules was demonstrated to be significantly larger than that of patients without the nodules, which was consistent with the suggestion that the formation of the siderotic nodules was interrelated with portal hypertension, as well as with portal hypertension resulting in splenomegaly, congestion and micro-hemorrhage in the spleen. However, the number of siderotic nodules did not always correspond with the increases in splenic volume, which may suggest that there are additional contributory mechanisms and interrelated factors.

Piccin *et al* (2) observed that patients with SCA and GGBs had low platelet counts, which was consistent with the results of the present study. This indicated that the presence of splenic siderotic nodules corresponded with the occurrence of thrombocytopenia in patients with liver cirrhosis. The mechanism of thromobocytopenia is complicated and disputed. At present, the suggestion is that hypersplenism, decreased thrombopoietin (TPO) synthesis and bone suppression result in thrombocytopenia in patients with liver cirrhosis (20–23). Thrombocytopenia may induce and aggravate hemorrhage; thus, it was proposed that, in addition to portal hypertension, thrombocytopenia may be an independent or coordinated cause of splenic siderotic nodules in patients with liver cirrhosis.

There were significant differences in reserved liver function between patients with and without splenic siderotic nodules, which demonstrated that the siderotic nodules were interrelated with liver function. This result contrasted with that described in a previous study by Laurent *et al* (18). It is likely that the poor reserved liver function decreased the synthesis of coagulation factor and TPO, which induced hemorrhages, including splenic hemorrhage.

In conclusion, the MRI ESWAN sequence was a sensitive method of detecting siderotic nodules of the spleen in patients with liver cirrhosis. A higher prevalence of splenic siderotic nodules (72.7%) was revealed using this sequence than with the sequence used in previous studies. The presence of splenic siderotic nodules was consistent with the occurrence of hypersplenism; therefore, the presence of splenic siderotic nodules may be applied for the diagnosis of hypersplenism. The formation of these nodules has been suggested to be attributed to portal hypertension, which was consistent with the results from the present study. In addition, it was suggested that an additional potential cause for the formation of the nodules may be thrombocytopenia. Moreover, it was observed that the presence of siderotic nodules was interrelated with reserved liver function in patients with liver cirrhosis.

There were two limitations in this study, including the small sample size. Additional investigations with large groups of patients are required to confirm the results of the present study. In addition, the present study did not refer to the results of splenic pathology, although it has been recognized in previous studies that images obtained using MRI are sufficient to achieve a specific diagnosis.

## Figures and Tables

**Table I.** Patient data (n=33).

Factor	Value
Mean age (years)	54
Gender	
Males (n)	25
Females (n)	8
Etiology	
Hepatitis (n)	30
Alcohol (n)	2
PSC (n)	1
Liver function: Child-Pugh	
A (n)	22
B (n)	10
C (n)	1
Blood laboratory examination	
Thrombocytopenia (n)	16
Leukopenia (n)	15
Erythropenia (n)	7
Hypohemoglobin (n)	5
Splenomegaly (n)	30
Splenic ESWAN images: Grades	
A (n)	9
B (n)	10
C (n)	14

PSC, primary sclerosing cholangitis; ESWAN, enhanced-susceptibility weighted angiography.

**Table II.** Distribution of the splenic ESWAN image grades between the hypersplenism and non-hypersplenism subgroups.

Image grade	Subgroup		Total (n)
	Hypersplenism	Non-hypersplenism	
A (n)	0	9^a^	9
B (n)	2	8^a^	10
C (n)	14	0^a^	14
Total (n)	16	17^a^	-

a P<0.001 compared with the hypersplenism subgroup. ESWAN, enhanced-susceptibility weighted angiography.

**Table III.** Prevalence of grade C images in the hypersplenism and non-hypersplenism subgroups.

Subgroup	Grade C (n)	Other grades (n)	Total (n)
Hypersplenism	14	2	16
Non-hypersplenism	0^a^	17	17
Total (n)	14	19	33

a P<0.001 compared with the hypersplenism subgroup.

**Table IV.** Distribution of Child-Pugh scores between patients with and without siderotic nodules (SNs).

Child-Pugh score	SN (−)	SN (+)	Total (n)
A (n)	8	14^a^	22
B (n)	1	9^a^	10
C (n)	0	1^a^	1
Total (n)	9	24	33

a P<0.001 compared with patients without SNs.

**Table V.** Distribution of the serum albumin scores between patients with and without siderotic nodules (SNs).

Albumin score	SN (−)	SN (+)	Total (n)
1 (n)	7	10^a^	17
2 (n)	2	13^a^	15
3 (n)	0	1^a^	1
Total (n)	9	24	33

a P<0.001 compared with patients without SNs.
